# NRG1 knockdown rescues PV interneuron GABAergic maturation deficits and schizophrenia behaviors in fetal growth restriction mice

**DOI:** 10.1038/s41420-022-01271-3

**Published:** 2022-12-02

**Authors:** Jianfeng Dong, Wen Chen, Nana Liu, Shujuan Chang, Wei Zhu, Jiuhong Kang

**Affiliations:** Clinical and Translational Research Center of Shanghai First Maternity and Infant Hospital, Shanghai Key Laboratory of Signaling and Disease Research, Frontier Science Center for Stem Cell Research, National Stem Cell Translational Resource Center, School of Life Sciences and Technology, Institute for Advanced Study, Tongji University, Shanghai, China

**Keywords:** Schizophrenia, Spatial memory

## Abstract

Schizophrenia is a highly debilitating mental disorder, those who experienced fetal growth restriction (FGR) in the early stage of life have a greater probability of schizophrenia. In this study, FGR mice showed hyperactivity in locomotor activity test, sociability dysfunction in three chamber test and nesting social behavior tests, cognition decline in Morris water maze and impaired sensory motor gating function in prepulse inhibition test. Mechanistic studies indicated that the number of parvalbumin (PV) interneuron was significantly reduced in FGR mouse media prefrontal cortex (mPFC). And the mRNA and protein level of neuregulin 1(NRG1), which is a critical schizophrenia gene, increased significantly in FGR mouse mPFC. Furthermore, NRG1 knockdown in FGR mouse mPFC improved PV interneuron GABAergic maturation and rescued schizophrenia behaviors including hyperactivity, social novelty defects, cognition decline, and sensorimotor gating deficits in FGR mice. This study indicates that mPFC NRG1 upregulation is one of the main causes of FGR-induced schizophrenia, which leads to significant reduction of PV interneuron number in mPFC. NRG1 knockdown in mPFC significantly rescues schizophrenia behaviors in FGR mouse. This study thus provides a potential effective therapy target or strategy for schizophrenia patients induced by FGR.

## Introduction

Schizophrenia is a severe and disabling mental disorder with a prevalence of around 1% worldwide [1]. It accounts for 3% of the total economic burden of human disease [2]. Besides the role of genetics in the etiology of schizophrenia [3, 4], non-genetic factors like the intra-uterine environment also contributes to this disorder [3, 5]. The important role of intra-uterine environment in the etiology of schizophrenia is in accord with schizophrenia’s neurodevelopmental origin [6]. Epidemiology investigation demonstrates that, schizophrenia is closely related to early-life complications (ELCs), which are adverse events that occur during pregnancy and labor, at delivery, and in neonatal life [7, 8]. Meta-analysis of this study reveals that ELCs increase risk by 1.5- to 2-fold, a greater effect than any common genetic variant in schizophrenia progression [8]. Fetal growth restriction (FGR) is an important disease in ELCs, which affects 6% of pregnant women worldwide [9], and is linked to high perinatal mortality and morbidity [10]. Epidemiological studies demonstrate that people with a history of FGR would double the prevalence of suffering from schizophrenia in adulthood [8, 11, 12], while the underling mechanism is unclear.

Schizophrenia is characterized by positive, negative and cognitive symptoms. Although positive symptoms are the most noticeable disease manifestation, cognitive symptoms are perhaps the most distinctive [13]. Studies indicate that inhibitory function deficits in the prefrontal cortex of schizophrenia patients might lead to cognitive disturbances [14–16]. Indeed, cortical inhibition impairments have been reported both in vivo and in post-mortem brain tissue from patients with schizophrenia [17, 18], and cognitive functions such as working memory depends on interneuron function [19, 20]. Only certain classes of cortical interneuron seem to be impaired in schizophrenia. In particular, parvalbumin (PV) interneurons in the prefrontal cortex of schizophrenia patients show decreased expression of glutamic acid decarboxylas1(GAD1) [21, 22], which impairs the inhibitory function of PV interneurons. However, it is unclear whether PV interneuron is the main cytological mechanism in FGR induced schizophrenia.

An increasing body of evidence suggests that variation in genes that control the development and maturation of PV interneurons such as NRG1-ErbB4 signaling [23–25], DISC1 [26, 27], GRIN1 [28] and DTNBP1 [29], might confer susceptibility to schizophrenia [30]. NRG1 is a family of trophic factors with an epidermal growth factor (EGF)-like domain [31, 32], and is critical in neural development and synaptic plasticity. Besides, it also plays an important role in the transformation of high-risk schizophrenia [33, 34]. NRG1 and its receptor ErbB4 have been linked to abnormal cortical function in schizophrenia [35, 36]. Studies demonstrate that NRG1 is significantly upregulated in prefrontal cortex and hippocampus of schizophrenia patients [37–39]. High expression of NRG1 in hippocampal pyramidal neurons induces schizophrenic symptoms, leading to cognitive decline and social behavior defects [40]. Besides, researches also show that NRG1-ErbB4 signaling plays a critical role in GABAergic circuit maturation in cerebral cortex [23]. However, whether NRG1 upregulation contributes to GABAergic maturation defects in FGR induced schizophrenia is still unclear.

In this study, we proved that NRG1 was pivotal in FGR induced schizophrenia and NRG1 knockdown in mPFC rescued schizophrenia symptoms in FGR mice. Besides, our results also demonstrated that mPFC NRG1 knockdown rescued FGR induced schizophrenia symptoms by improving GABAergic maturation of PV interneurons. These observations reveal a fundamental role of NRG1 in balancing prefrontal cortex inhibitory function, identify PV interneurons as a cellular target of NRG1-ErbB4 signaling in cognition and complex behaviors relevant to FGR-induced schizophrenia.

## Results

### FGR leads to schizophrenia behavioral defects

**Fig. 1:**
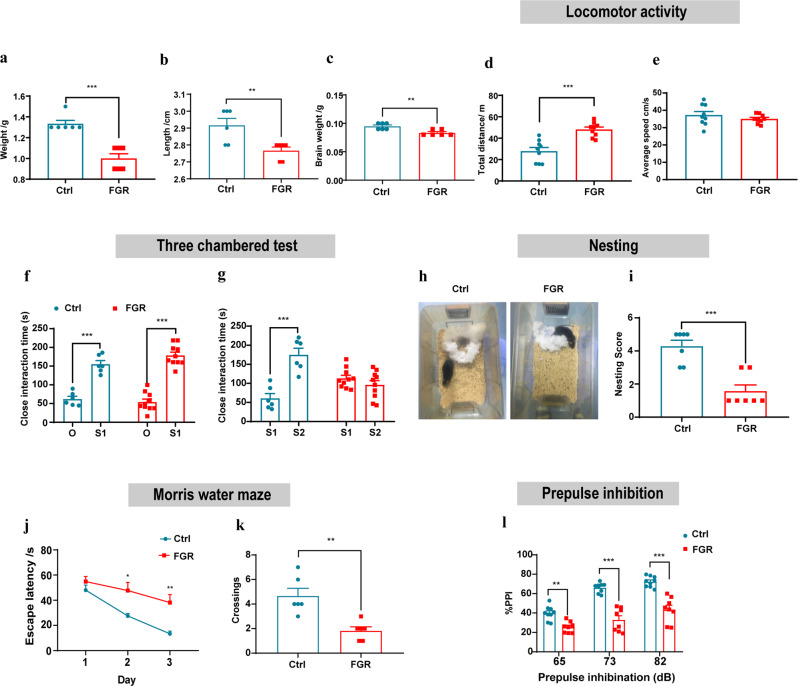
FGR leads to schizophrenia behavioral defects. **a**–**c** Weight, length and brain weight of neonatal mice. *n* = 6 in each group, weight of control and FGR mouse, unpaired *t* test, *P* < 0.001; length of control and FGR neonatal mice, unpaired *t* test, *P* < 0.01; brain weight of control and FGR neonatal mice, unpaired *t* test, *P* < 0.01. **d**, **e** Locomotor activity of control (*n* = 10) and FGR (*n* = 10) mice. **d** Total distance, unpaired *t* test, *P* < 0.001. **e** Average speed, unpaired *t* test, *P* = 0.328. **f**, **g** Three chamber test of control (*n* = 6) and FGR (*n* = 10) mice. **f** Time spent on close interaction with an object (O) versus stranger mouse (S1) in the social approaching test. Two-way ANOVA, *P* Control < 0.001; *P* FGR < 0.001. **g** Time spent on close interaction with a familiar mouse (S1) versus stranger mouse (S2) in the social novelty test. Two-way ANOVA, *P* control < 0.001; *P* FGR = 0.455. **h**, **i** Nest building test of control (*n* = 7) and FGR (*n* = 7) mice. **h** Representative pictures of control and FGR nest. (**i**) Nest building score, unpaired *t* test, *P* < 0.001. **j**, **k** MWM performance of control (*n* = 6) and FGR (*n* = 6) mice. **j** Spatial acquisition performance. Two-way ANOVA, *P* day1 = 0.650; *P* day2 = 0.011; *P* day3 = 0.002. **k** Platform crossing, unpaired *t* test of target quadrant, *P* = 0.002. **l** Prepulse inhibition test of control (*n* = 9) and FGR (*n* = 8) mice. Two-way ANOVA, *P* 65db = 0.003; *P* 73db < 0.001; *P* 82db < 0.001. The results are expressed as mean ± SD.; *P < 0.05, **P < 0.01, and ***P < 0.001.

FGR is mainly caused by abnormal uterine function. In this study, pregnant mice were intraperitoneally injected of dexamethasone to induce uterine function impairment. FGR neonatal mice showed significantly decreased body weight, brain weight, and crown-rump length (Fig. 1a–c). A series of schizophrenia related behavioral tests were carried out, including locomotor activity test, social behavior tests, cognitive behavior test and sensory motor gating test. In locomotor activity, the total distance was significantly increased FGR mice, while the average speed was comparable between two groups (Fig. 1d, e). Social proximity and social novelty were mainly detected by three chamber social behavior test (Fig. 1f, g). In social approaching stage, both FGR and control mice showed considerable sociability (Fig. 1f). In social novelty stage, when the second stranger mouse was introduced, the exploration time of FGR mice to the second stranger mouse was significantly reduced compared with control mice (Fig. 1g). In nesting experiment, control mice completed the nesting task within 24 h, while FGR mice showed poor performance in this task (Fig. 1h). The nesting score of FGR mice was significantly reduced compared with control mice (Fig. 1i). Three chamber behavior test and nesting experiment results showed that the sociability was significantly impaired in FGR mice. Then, we tested the cognition of FGR and control mice, as schizophrenic patients showed significant decline in learning and memory. FGR led to significant defects in spatial learning and 24 h short-term memory in Morris water maze (Fig. 1j, k). Prepulse inhibitory is a classical test in evaluating the sensory gating function in schizophrenia research. Results showed that the PPI of control mice increased gradually as the elevation of pre-stimulation, while the PPI was significantly reduced in FGR mice, which indicated profound sensory gating defects in FGR mice (Fig. 1l). All the above behavioral test results indicate that FGR leads to schizophrenia behavioral defects.

### FGR results in mPFC PV interneuron GABAergic maturation defects

**Fig. 2:**
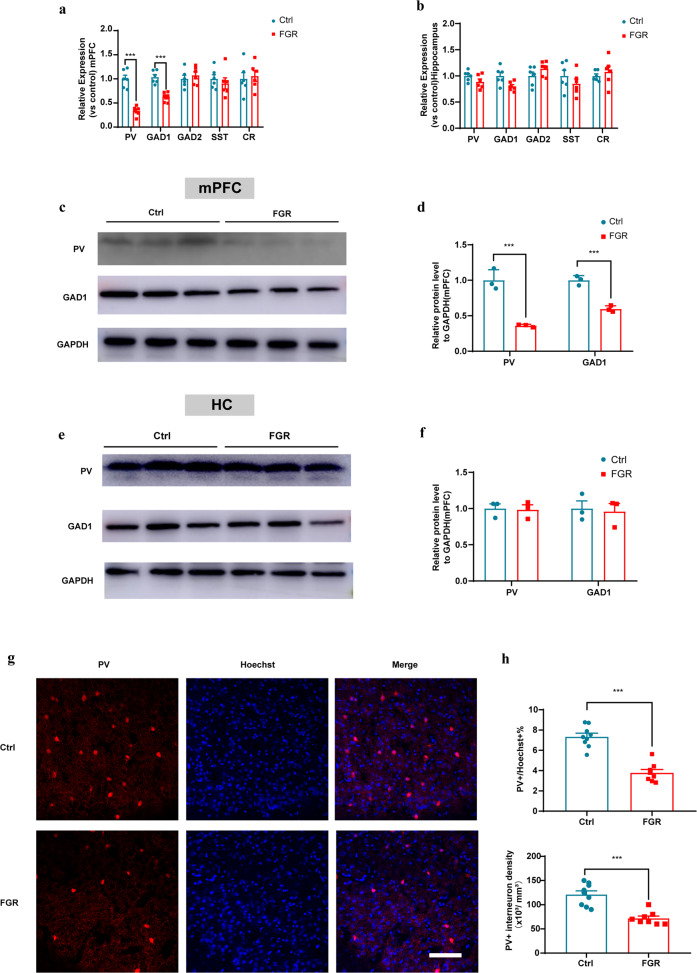
FGR results in mPFC PV interneuron GABAergic maturation defects. **a** PV, GAD1, GAD2, SST and CR mRNA levels in the mPFC of control (*n* = 6) and FGR (*n* = 6) mice. PV: unpaired *t* test, *P* < 0.001. GAD1: unpaired *t* test, *P* < 0.001. GAD2: unpaired *t* test, *P* = 0.496. SST: unpaired *t* test, *P* = 0.529. CR: unpaired *t* test, *P* < 0.001. **b** PV, GAD1, GAD2, SST, and CR mRNA levels in the hippocampus of control (*n* = 6) and FGR (*n* = 6) mice. PV: unpaired *t* test, *P* = 0.054. GAD1: unpaired *t* test, *P* = 0.159. GAD2: unpaired *t* test, *P* = 0.166. SST: unpaired *t* test, *P* = 0.313. CR: unpaired *t* test, *P* = 0.491. **c**, **d** Representative blots and statistical analysis of PV and GAD1 protein in the mPFC. **e**, **f** Representative blots and statistical analysis of PV and GAD1 protein in the hippocampus. **g** Representative images of mouse mPFC region immunostained with PV and Hoechst antibodies. Scale bar = 100 μm. **h** Statistical analysis of PV interneuron percentage and density in mPFC region, *n* = 9 in control group, *n* = 8 in FGR group. The results are expressed as mean ± SD.; *P < 0.05, **P < 0.01 and ***P < 0.001.

PV interneuron dysfunction plays a critical role in schizophrenia progression [41, 42]. We detected the maturation of PV interneuron in hippocampus and mPFC regions of FGR and control mice. QPCR results showed that the mRNA levels of PV and GAD1 were significantly decreased in mPFC of FGR mice, while GAD2 were comparable in FGR and control groups (Fig. 2a, b). And the mRNA levels of SST and CR, another two important interneuron marker genes, were comparable between FGR and control mice hippocampus and mPFC (Fig. 2a, b). Western blot results indicated that the protein levels of PV and GAD1 of FGR mice mPFC region decreased significantly (Fig. 2c, d and whole membrane image 1), while these were comparable in hippocampus (Fig. 2e, f and whole membrane image 2). Meanwhile, immunostaining results also proved that the percentage and density of PV interneurons were significantly decreased in mPFC of FGR mice (Fig. 2g, h), while these were comparable in hippocampal DG, CA1 and CA3 regions between FGR and control mice (Supplementary Fig. 1). The above results indicated that the GABAergic maturation of PV interneurons was significantly impaired in the mPFC region of FGR mice.

### FGR leads to upregulation of NRG1 in mPFC

**Fig. 3:**
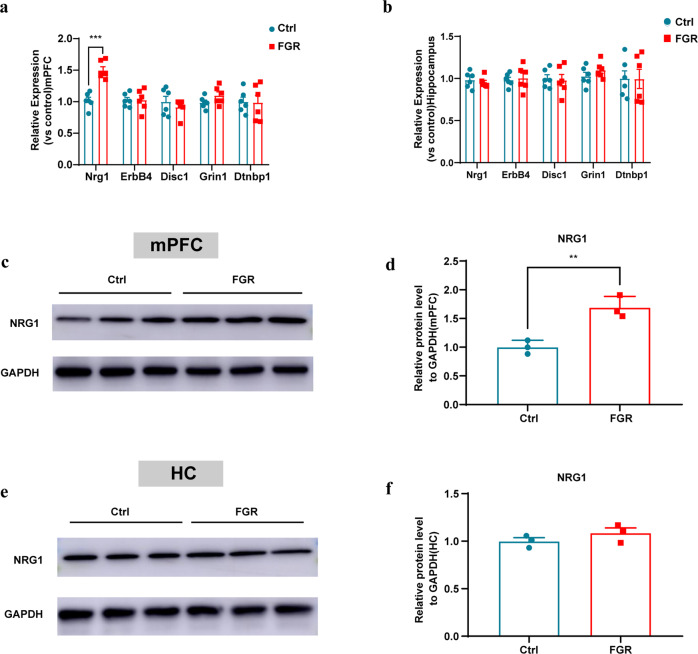
FGR leads to upregulation of Nrg1 in mPFC. **a** Nrg1, ErbB4, Disc1, Grin1 and Dtnbp1 mRNA levels in the mPFC of control (*n* = 6) and FGR (*n* = 6) mice. Nrg1: unpaired *t* test, *P* < 0.001. ErbB4: unpaired *t* test, *P* = 0.982. Disc1: unpaired *t* test, *P* = 0.423. Grin1: unpaired *t* test, *P* = 0.134. Dtnbp1: unpaired *t* test, *P* = 0.925. **b** Nrg1, ErbB4, Disc1, Grin1 and Dtnbp1 mRNA levels in the hippocampus of control (*n* = 6) and FGR (*n* = 6) mice. Nrg1: unpaired *t* test, *P* = 0.528. ErbB4: unpaired *t* test, *P* = 0.755. Disc1: unpaired *t* test, *P* = 0.809. Grin1: unpaired *t* test, *P* = 0.252. Dtnbp1: unpaired *t* test, *P* = 0.973. **c**, **d** Representative blots and statistical analysis of NRG1 protein in the mPFC from control and FGR mice. **e**, **f** Representative blots and statistical analysis of NRG1 protein in the hippocampus from control and FGR mice. The results are expressed as mean ± SD.; *P < 0.05, **P < 0.01, and ***P < 0.001.

NRG1 is fundamental in schizophrenia progression [35, 36], and was found significantly upregulated in the brain of schizophrenic patients [37–39]. In this study, we found that the mRNA level of Nrg1 was increased in mPFC of FGR mice (Fig. 3a), while the mRNA levels of ErbB4, Disc1, Grin1 and Dtnbp1 were comparable in both mPFC and hippocampus between two groups(Fig. 3a, b). Western blot results also indicated that the expression of NRG1 protein increased significantly in mPFC of FGR mice (Fig. 3c, d and whole membrane image 3), while no significant change was observed in hippocampus (Fig. 3e, f and whole membrane image 4).

Moreover, the postnatal dynamic mRNA and protein expression of PV and NRG1 were assessed during P0-P60. FGR and control mice were sacrificed at 0, 7, 30 and 60 days after birth. Their hippocampus and mPFC were extracted to detect the mRNA and protein level of PV and NRG1. The mRNA level of PV in the mPFC of FGR mice was significantly decreased from the 30th day after birth compared with control group (Supplementary Fig. 2a). While in hippocampus, the mRNA level of PV was comparable between two groups (Supplementary Fig. 2b). The protein level of PV in both mPFC and hippocampus of FGR mice was significantly lower than control mice from the 30th day after birth (Supplementary Fig. 2e, f and whole membrane image 5, 6). Besides, in FGR mice mPFC, the mRNA and protein level of NRG1 increased significantly from the 7th day after birth (Supplementary Fig. 2c, e, and whole membrane image 5). While in hippocampus, the level of NRG1 was comparable between two groups (Supplementary Fig. 2d, f, and whole membrane image 6). Hence, we made an assumption that the significant upregulation of NRG1 in FGR mice mPFC may be one main cause of schizophrenia behavior induced by FGR.

### NRG1 knockdown improves FGR PV interneurons GABAergic maturation

**Fig. 4:**
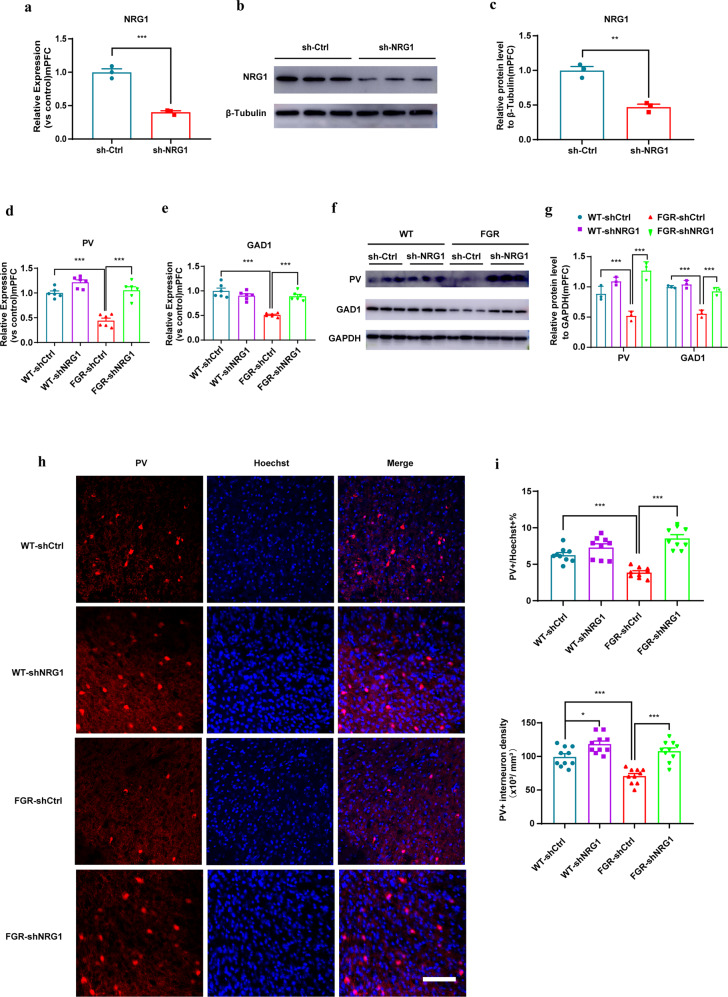
NRG1 mPFC knockdown rescues FGR induced PV interneuron GABAergic maturation defects. **a** Nrg1 mRNA QPCR test. *n* = 3 in each group, unpaired *t* test, *P* < 0.001. **b**, **c** NRG1 protein western blot test and statistical analysis. *n* = 3 in each group, unpaired *t* test, *P* < 0.01. **d** PV mRNA levels, *n* = 6 in each group. One-way ANOVA, WT- shCtrl vs FGR- shCtrl*, P* < 0.001; FGR- shCtrl vs FGR- shNRG1, *P* < 0.001. **e** GAD1 mRNA levels, *n* = 6 in each group. One-way ANOVA, WT- shCtrl vs FGR- shCtrl*, P* < 0.001; FGR- shCtrl vs FGR- shNRG1, *P* < 0.001. **f**, **g** PV and GAD1 protein western blot test and statistical analysis. *n* = 3 in each group. One-way ANOVA. PV: WT- shCtrl vs FGR- shCtrl*, P* < 0.001; FGR- shCtrl vs FGR- shNRG1, *P* < 0.001. GAD1: WT- shCtrl vs FGR- shCtrl*, P* < 0.001; FGR- shCtrl vs FGR- shNRG1, *P* < 0.001. **h** Representative images of mouse mPFC region immunostained with PV and Hoechst antibodies. Scale bar = 100 μm. **i** Statistical analysis of PV interneuron percentage and density in mPFC, *n* = 9 in each group. One-way ANOVA, WT- shCtrl vs FGR- shCtrl*, P* < 0.001; FGR- shCtrl vs FGR- shNRG1, *P* < 0.001. The results are expressed as mean ± SD.*;* *P < 0.05, **P < 0.01, and ***P < 0.001.

To verify the importance of high mPFC NRG1 level in FGR induced schizophrenia progression, we constructed a shNRG1 lenti-virus. shNRG1 was driven by U6. One week after we injected the virus into the mPFC of mice, the mPFC was extracted to detect the mRNA level of Nrg1. QPCR results showed that Nrg1 was significantly knockdown in shNRG1 group (Fig. 4a). Western blot results also indicated that the protein level of NRG1 was significantly reduced in shNRG1 mouse group (Fig. 4b, c and whole membrane image 7). Then, we stereotactically injected shNRG1 lenti-virus or shCtrl lenti-virus to the mPFC of control and FGR mice. One week after injection, the mRNA level of PV and GAD1was detected. QPCR results showed that, the mRNA level of PV and GAD1 were significantly upregulated in FGR mice mPFC (Fig. 4d, e). Western blot results showed that, the protein level of PV and GAD1 were also significantly upregulated in FGR mice mPFC (Fig. 4f, g and whole membrane image 8). Moreover, immunostaining of PV in the mPFC region showed that the percentage and density of PV interneurons increased significantly in FGR-shNRG1 group compared with FGR-shCtrl mice (Fig. 4h, i). These results proved that NRG1 knockdown in mPFC improved the PV interneuron GABAergic maturation of FGR mice.

### NRG1 mPFC knockdown rescues FGR induced schizophrenia behavior

**Fig. 5:**
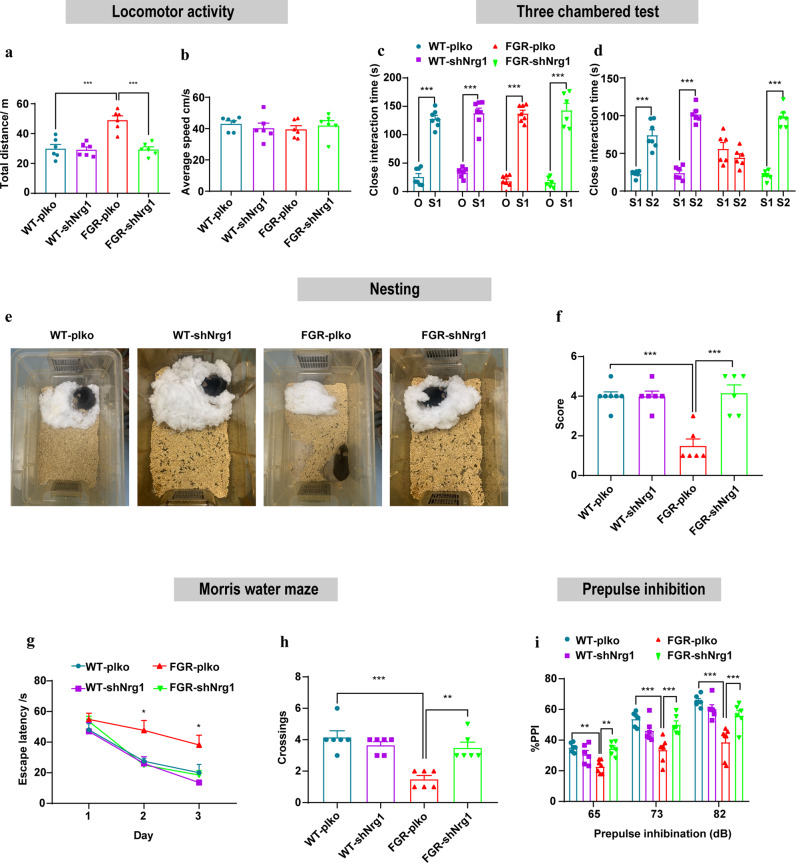
NRG1 mPFC knockdown rescues FGR induced schizophrenia behavior. **a**, **b** Locomotor activity, *n* = 6 in each group. **a** Total distance. One-way ANOVA, WT-shCtrl vs FGR- shCtrl*, P* < 0.001; FGR- shCtrl vs FGR-shNRG1, *P* < 0.001. **b** Average speed. **c** Time spent on close interaction with an object (O) versus stranger mouse (S1) in the social approaching test. Two-way ANOVA*, P* WT- shCtrl < 0.001*; P* WT-shNRG1 < 0.001; *P* FGR- shCtrl < 0.001; *P* FGR-shNRG1 < 0.001. **d** Time spent on close interaction with a familiar mouse (S1) versus stranger mouse (S2) in the social novelty test. Two-way ANOVA, interaction time of each group spent with S2 than S1 (*P* WT- shCtrl < 0.001; *P* WT-shNRG1 < 0.001; *P* FGR- shCtrl = 0.402; *P* FGR-shNRG1 < 0.001). **e**, **f** Nest building test of WT- shCtrl (*n* = 7), WT-shNRG1 (*n* = 6), FGR- shCtrl (*n* = 6) mice, FGR-shNRG1 (*n* = 6) mice. **e** Representative pictures of nests. **f** Nesting score. One-way ANOVA,WT- shCtrl vs FGR- shCtrl*, P* < 0.001; FGR- shCtrl vs FGR-shNRG1, *P* < 0.001. **g**, **h** MWM performance, *n* = 6 in each group. **g** Spatial acquisition performance. Two-way ANOVA, day2: WT- shCtrl vs FGR- shCtrl, *P* = 0.01; FGR- shCtrl vs FGR-shNRG1, *P* = 0.003. day3: WT- shCtrl vs FGR- shCtrl, *P* = 0.02; FGR- shCtrl vs FGR-shNRG1, *P* = 0.01). **h** Platform crossing. One-way ANOVA, WT- shCtrl vs FGR- shCtrl*, P* < 0.001; FGR- shCtrl vs FGR-shNRG1, *P* < 0.001. **i** Prepulse inhibition test of WT- shCtrl (*n* = 6), WT*-*shNRG1 (*n* = 6), FGR- shCtrl (*n* = 7) mice, FGR-shNRG1 (*n* = 6) mice. PPI of four mice groups. Two-way ANOVA, 65db: WT- shCtrl vs FGR- shCtrl, *P* = 0.004; FGR- shCtrl vs FGR-shNRG1, *P* = 0.008. 73db: WT- shCtrl vs FGR- shCtrl, *P* < 0.001; FGR- shCtrl vs FGR-shNRG1, *P* < 0.001. 82db: WT- shCtrl vs FGR- shCtrl, *P* < 0.001; FGR- shCtrl vs FGR-shNRG1, *P* < 0.003. The results are expressed as mean ± SD.; *P < 0.05, **P < 0.01, and ***P < 0.001.

One week after lenti-virus mPFC stereotactic injection, we performed a variety of schizophrenia behavioral tests. Results showed that mPFC NRG1 knockdown significantly reduced the total distance of locomotor activity in FGR mice group, while the average speed was not affected (Fig. 5a, b). In addition, in three chamber social behavior test, the sociability of four groups of mice was not altered in social approaching stage, while in social novelty stage, the communication and exploration time with stranger mice was significantly upregulated after mPFC NRG1 knockdown in FGR mice (Fig. 5c, d). Meanwhile, mPFC NRG1 knockdown also significantly improved the nesting score of FGR mice (Fig. 5e, f). These two behavior tests showed that mPFC NRG1 knockdown rescued the social defects in FGR mice. Besides, Morris water maze results showed that mPFC NRG1 knockdown significantly improved the spatial learning and 24 h short-term memory of FGR mice (Fig. 5g, h). Finally, the prepulse inhibition test results showed that mPFC NRG1 knockdown significantly upregulated the PPI and rescued the sensorimotor gating function of FGR mice (Fig. 5i). Meanwhile, the PPI of control mice was not altered after mPFC NRG1 knockdown. Collectively, these results indicated that mPFC NRG1 knockdown rescued schizophrenia behaviors in FGR mice.

## Discussion

Schizophrenia is a complex disabling disorder, genetic and environmental factors are involved in its etiology. DISC1 [43, 44] and 22q11.2 microdeletion [45, 46] are established genetic factors of schizophrenia. Intra-uterine environment impairment induced by prenatal infection or malnutrition [3, 5] is included in non-genetic factors. Animal studies show that exposure to environmental insults in utero leads to altered response to stress postnatally, which results in abnormal brain development and behavior [47, 48], but the mechanism remains unclear. In this study, we found that the leading cause of FGR-induced schizophrenia was the significant upregulation of NRG1 in mPFC, which impaired PV interneuron GABAergic maturation and resulted in subsequent schizophrenia behaviors in mice.

PV interneuron plays a key role in the pathogenesis of schizophrenia [21, 22]. Studies propose two possible mechanisms that may explain the role of PV interneurons in schizophrenia. First, deficits of PV interneurons lead to disinhibition of pyramidal neurons, and results in hyperactivity and cognition decline in schizophrenia patients [49]. Second, PV interneuron plays a critical role in cortical gamma-band synchrony, and the dysfunction of PV interneurons leads to logic reasoning impairment and working memory deficits [50]. In this study, we found that FGR led to cognition decline, attention deficits, and executive function impairment, which was in accord with the cortical disinhibition symptoms of schizophrenia patients. The dysfunction of PV interneurons in mPFC leads to significant cortical disinhibition, which may result in hyperactivity, social ability deficiency, and sensorimotor gating deficits. Meanwhile, PV interneuron impairment may cause the dysfunction of pyramidal neurons and hence lead to memory impairment.

NRG1 is a fundamental gene in the maturation of GABAergic interneurons. NRG1 knockout in mouse MGE region leads to significant GABAergic dysfunction in mice model. Our results indicate that the GABAergic maturation of PV interneuron, which is critical in cortical disinhibition, is significantly impaired in FGR mouse mPFC. Improving the GABAergic maturation of PV interneurons through NRG1 knockdown in mPFC significantly rescued the schizophrenia behaviors in FGR mice. Recent studies have found increased NRG1 signaling in the PFC of schizophrenia patients, which stimulates GABA transmission [51]. This leads to hypofunction of the glutamatergic pathway, reduced glutamatergic transmission, and plasticity in the brains of patients with schizophrenia [52, 53]. In our study, we found that FGR resulted in reduced expression of GAD1 and PV in mouse mPFC, which could be rescued by NRG1 knockdown. As NRG1 regulates GABAergic transmission, it is possible that the upregulation of NRG1 compensates for reduced GAD1 and PV activity. This is the first time that the specific brain region and cellular target of FGR-induced schizophrenia are determined.

Antipsychotics like clozapine and risperidone are effective against positive symptoms, while the hematological and metabolic side effects occur frequently [54, 55]. Meanwhile, FGR leads to metabolic diseases including heart disease [56], diabetes [57] and insulin resistance [58]. The side effects of psychotropic drugs may aggravate the process of metabolic diseases induced by FGR. Besides, antipsychotic drugs could hardly relieve negative symptoms and cognition decline in schizophrenia patients. In this study, we found that mPFC NRG1 knockdown rescued cognition decline, social novelty defects and sensorimotor gating impairment in FGR mice without hematological and metabolic side effects. This treatment is probably an important supplement for existing antipsychotics for FGR-induced schizophrenia patients.

Collectively, our study provides direct evidence that NRG1 is a critical molecule that bridges PV interneuron GABAergic maturation and schizophrenia behaviors in offspring with FGR. Besides, we determine that mPFC is the main brain region in FGR-induced schizophrenia. We propose that mPFC NRG1 knockdown could be a suitable therapy for alleviating schizophrenia behaviors in FGR.

## Materials and methods

### Animals

FGR model was established by intraperitoneal injection of dexamethasone during perinatal period. On the 14.5th day, the success thrombus test was calculated as 0.5 day. 0.1 mg/kg dexamethasone was injected intraperitoneally for 5 consecutive days. In this study, 3-month-old offspring male mice were used for behavioral tests. Five mice were fed in each cage, and there was sufficient water and feed in the mouse cage. The feeding environment was set with lighting time from 7:00 am to 7:00 pm every day, and the lights were turned off at other time. After the operation, mice were raised separately in the same environment. All mice were raised separately according to their sex, age and species. 6–9 mice were included in each group for the sake of animal death during behavior tests and stereotactic injection. All animals that participate in the experiment were included for analysis, unless animal death. There was no randomization used in our study. All animal treatment processes were recognized by the animal management and use association of Tongji University.

### Behavior

All behavioral experiments were conducted in the light environment from 9:00 am to 6:00 pm. Mice were permitted to rest for 2 weeks between different behavioral tests. Up to four behavioral tests were performed in a batch of mice.

### Locomotor activity

The locomotor activity test was performed as previously described [59]. The open-field test was carried out in a computer-operated detecting and analysis apparatus (Med Associates). Each mouse was placed in the field of 27.31 × 27.31 cm (*L* × *W*) with a dimly lit background for 30 min of spontaneous exploration. Average velocity and total distance traveled in the whole arena were recorded in this test.

### Three-chamber social interaction test

Sociability and social novelty tests were performed as previously described [60]. Sociability and social novelty were analyzed in an arena (60 × 60 cm) with two equidistant clear Plexiglas cylinders (each 7 cm in diameter, 12 cm tall). The cylinders had multiple holes (1 cm in diameter) to allow the auditory, visual and olfactory interaction between the stimulus mouse placed inside and the test mouse outside of the cylinder. Between trials, the apparatus and cylinders were cleaned with 75% ethanol and dried before testing the next mouse. Time sniffing the social and non-social cylinders was operationally defined as the time during which the test mouse made direct nose-to-cylinder contact with the social and non-social cylinders, respectively. This was captured and recorded with Sony digital video camera mounted above the chamber. The paradigm consisted in a three-stage procedure: During habituation phase, the testing mouse was allowed to initially explore the apparatus with the cylinders for 10 min. In social approaching phase, an unfamiliar sex-matched mouse was placed in one of the cylinder, maintaining the other empty. Finally, in social novelty test, another unfamiliar mouse was introduced to the other empty cylinder. In the preference for social novelty test, preference score was calculated by subtracting the time spent sniffing the two stranger mice.

### Nest building test

Nest building test was performed as previously described [61]. To test nest building ability of mice, one square piece of nesting material made of cotton fiber (5 × 5 cm) was introduced to a cage in which mouse was individually housed. Pictures of the nests were taken 24 h later with a Sony digital video camera. The quality of the nest was assessed using the following score criteria: 1_nestlet not noticeable touched, 2_nestlet partially torn up, 3_ mostly shredded but not identifiable nest site, 4_an identifiable but flat nest, 5_ a well-defined nest with walls.

### Prepulse inhibition (PPI) test

PPI test was performed as previously described [62], with minor modifications. Experiments were performed with sound attenuating test chambers (65 × 35 × 25 cm, *L* × *W* × *H*). Each chamber was equipped with a commercial startle reflex system (SR Lab). Each test session began with a 5 min acclimation period in the presence of 50 dB acoustic background noise followed by twelve 100 dB startle pulse (20 ms). In the subsequent 48 trails, the startle tone was either presented alone or after three levels of prepulse intensity (65, 72 and 83 dB, 20 ms) with a delay of 100 ms in a randomized order. The average inter trial between each trial was 30 s (range: 20–40 s). The average startle amplitude during the 100 ms following the onset of each startle stimulus was automatically recorded. Percentage of prepulse inhibition was calculated for each mouse at each prepulse stimulus intensity using the equation: PPI = 100 − [(prepulse/startle alone) × 100], where prepulse is the average startle response on trials in which there was a prepulse stimulus and startle alone is the average startle response on the trials in which the only startle stimulus was presented (excluding the first 12 trials of the session).

### Morris water maze

To assess spatial learning and memory, Morris water maze test was performed as described previously [63]. The Morris water maze was conducted in a circular pool (diameter, 120 cm). Tempera paint was added into the water until it became opaque. And a hidden platform (diameter, 10 cm) was placed 1.0 cm below the water. The water temperature was kept at ±1 °C from 22 °C. Distinct visual cues were hung on the privacy blinds. Mice were monitored using the Ethovision XT (Noldus Information Technology) video tracking system above the water tank and parameters were measured using Ethovision software. Mice were randomly put into the water from four learning positions and swam freely for 1 min. Mice were forced to stand on the platform for 20 s if they did not find and stand on the platform within 1 min. The trial was repeated four times for each mouse during each day of training. Three days of training were performed and a probe trial was conducted on the fourth day.

### Lenti virus preparation and stereotactic injection

Lv-plko was constructed in our lab. shNRG1 was driven by U6 promoter. All viruses were titered at about 1.0 × 10^8^ TU /ml. All viruses were stored at −80 °C, and used within 3 months. The sequences targeting NRG1 (GenBank Accession: NM-178591) that were used in the experiments were as follows: shCtrl: 5′-TTCTCCGAACGTGTCACGT-3′, shNRG1: 5′-GCAAACTGCTCCTAAACTT-3′. 293-FT cells were purchased from ATCC(IM-H053), and were recently tested for mycoplasma contamination.

Before stereotactic injection, mice were intraperitoneally injected with 1.25% phenobarbital for anesthesia. After that, the mouse head was fixed, the syringe was fixed, and the injection speed of the syringe was adjusted to 0.2 μl/min. The virus was injected at mPFC (anteroposterior (AP) + 1.9 mm, mediolateral (ML) + 0.4 mm, dorsoventral (DV, relative to dura −2.5 mm). About 0.2 μl virus solution was injected to each mPFC region in two hemispheres of mouse brain. After injection, waiting for 10 min before taking out the needle (to prevent liquid leakage). The mouse scalp was sutured and the mouse was placed on the heater until it awoke. Mice were fed separately for one week after virus injection, and then 5 mice were fed in a cage after the wound was completely healed.

### Western blot

Approximately 25 μg total protein was loaded per lane, which was then separated by SDS-PAGE and transferred to the membrane. The sealing solution is 1×TBST solution containing 3% BSA. Sealing at room temperature for 1 h. 1×TBST solution containing primary antibody was added and incubated on a shaking table at 4 °C overnight. Then, the membrane was washed with 1×TBST on a shaking table at room temperature for 5 min each time, 3 times. After that, 1×TBST solution containing secondary antibody was added and incubated on a shaking table at 4 °C for 2 h. Then the membrane was washed with 1×TBST on a shaking table at room temperature for 5 min each time, 3 times. The primary antibodies used for immunostaining were: rabbit anti-PV (1:1,000, Swant, PV27), mouse anti-NRG1(1:1,000, Santa Cruze, sc-393006), rabbit anti-GAD1(1:1,000, Millipore, MAB5406), rabbit anti-GAPDH (1:3,000, Bioworld, AP0013), rabbit anti-β-Tubulin (1:3,000, Invitrogen, 18-0093). The secondary antibodies used for immunostaining were: mouse HRP (1:3,000, Cell Signaling Technology, 7076 s), rabbit HRP (1:3,000, Cell Signaling Technology, 7074 s).

### Quantitative real-time PCR

Real-time polymerase chain reaction (PCR) was performed with SYBR-Green-based reagents (Invitrogen) using a CFX96 real-time PCR Detection system (Bio-Rad). The total RNA was extracted with TRIzol (Invitrogen) using a homogenizer according to the manufacturer’s protocol. The relative quantities of DNA fragments were calculated using the comparative CT method. The results were compared to a standard curve generated by serial dilutions of input DNA. Primers sequences were: ATCAAGAAGGCGATAGGAGCC and GGCCAGAAGCGTCTTTGTT for PV; CACAGGTCACCCTCGATTTTT and ACCATCCAACGATCTCTCTCATC for GAD1; TCCGGCTTTTGGTCCTTCG and ATGCCGCCCGTGAACTTTT for GAD2; ACCGGGAAACAGGAACTGG and TTGCTGGGTTCGAGTTGGC for SST; AGTACACCCAGACCATACTACG and GGCCAAGGACATGACACTCTT for CR; ACCAGCCATCTCATAAAGTGCG and GGCCAAGGACATGACACTCTT for NRG1; GTGCTATGGACCCTACGTTAGT and TCATTGAAGTTCATGCAGGCAA for ErbB4.

### Tissue processing and immunohistochemistry analysis

Mice were anaesthetized and transcardially perfused with 20 ml of 1 M phosphate-buffered saline (PBS). Hippocampal tissue was stored in 4% paraformaldehyde at 4 °C for 8 h then switched to 30% sucrose solution for an additional 48 h in preparation for brain sectioning. Serial coronal 40 µm sections of the hippocampus and mPFC (+1.8 mm to +1.95 mm from bregma) were collected using a freezing microtome (Leica, SM2010R) and stored in cryoprotective medium (40% PBS, 30% glycerol and 30% ethylene glycol) at −20 °C until immunostaining. Immunohistochemistry analysis was performed using standard techniques using free-floating sections. The primary antibody used for immunostaining was: rabbit anti-PV (1:5,000, Swant, PV27). All fluorescent secondary antibodies were diluted at a concentration of 1:1,000 and incubated with sections for 2 h at 4 °C. The fluorescent secondary antibody used for immunostaining was (1:1,000, Thermo Fisher Scientific). Nuclei was fluorescently labeled with Hoechst 33342 (1:2,000, Sigma-Aldrich, B2261). For quantification, overlapping images of mPFC and different regions of hippocampus on each section were captured by confocal microscopy in z stack. The density of PV interneurons was determined by dividing the total number of PV interneurons by the corresponding volume of mPFC or hippocampal regions.

### Statistics

All statistical data are from the results of more than three repeated experiments. The data shown in the figure is the mean ± standard deviation (mean ± SD). One way ANOVA analysis or two way ANOVA analysis is used for statistics. If *P* < 0.05, it is considered to have statistical difference, it is represented by ‘*’; If *P* < 0.01, it is indicated by ‘**’; If *P* < 0.001, it is indicated by ‘***’.

### Blind statement

The investigator was blinded to the group allocation during the experiment and/or when assessing the outcome.

## Supplementary information

Supplementary Figure

Full and uncropped western blots

## Data Availability

All data generated or analyzed during this study are included in this published article and its supplementary information files.
