# Supplementary material for: NRG1 knockdown rescues PV interneuron GABAergic maturation deficits and schizophrenia behaviors in fetal growth restriction mice
Source: Cell Death Discov. 2022 Dec 2;8:476. doi: 10.1038/s41420-022-01271-3 (PMC9718849; doi:10.1038/s41420-022-01271-3)
Supplement: Supplementary file 1 — Supplementary Figure [file 41420_2022_1271_MOESM1_ESM.docx]

**Supplementary Materials**

**NRG1 knockdown rescues PV interneuron GABAergic maturation deficits and schizophrenia behaviors in fetal growth restriction mice**

Jianfeng Dong^1^, Wen Chen^1^, Nana Liu^1^, Shujuan Chang^1^, Wei Zhu^1^, Jiuhong Kang^1*^

**This supplementary material includes two supplementary figures. Supplementary figure1 is the representative immunostaining pictures of PV interneurons in DG, CA1 and CA3 regions of control and FGR mice, and the statistical analysis of the percentage and density of PV interneurons. Supplementary figure 2 is the postnatal dynamic mRNA and protein expression of PV and NRG1 during P0-P60.**

**Supplementary Figure 1**


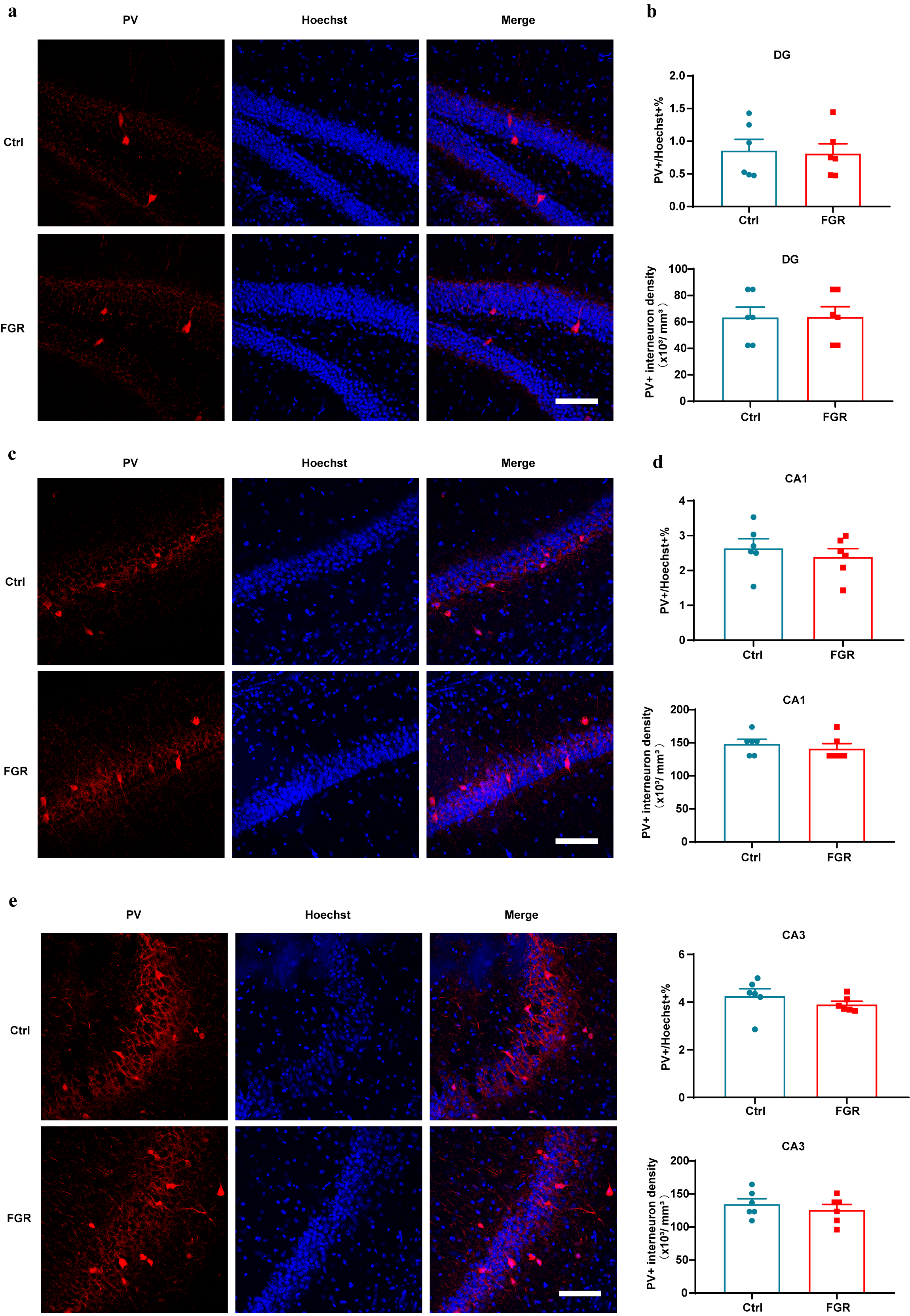


**Supplementary Figure 1 PV interneuron in hippocampus of control and FGR mice. (a**) Representative images of PV interneuron immunostaining with PV and Hoechst in DG region. Scare bar = 100 μm. (**b**) Statistical analysis of PV interneuron percentage and density in DG region, n = 6 in each group. **(c**) Representative images of PV interneuron immunostaining with PV and Hoechst in CA1 region. Scare bar = 100 μm. (**d**) Statistical analysis of PV interneuron percentage and density in CA1 region, n = 6 in each group. **(e**) Representative images of PV interneuron immunostaining with PV and Hoechst in CA3 region. Scare bar = 100 μm. (**f**) Statistical analysis of PV interneuron percentage and density in CA3 region, n = 6 in each group. The results are expressed as the mean ± SD.; **P* < 0.05, ***P* < 0.01 and ****P* < 0.001.

**Supplementary Figure 2**


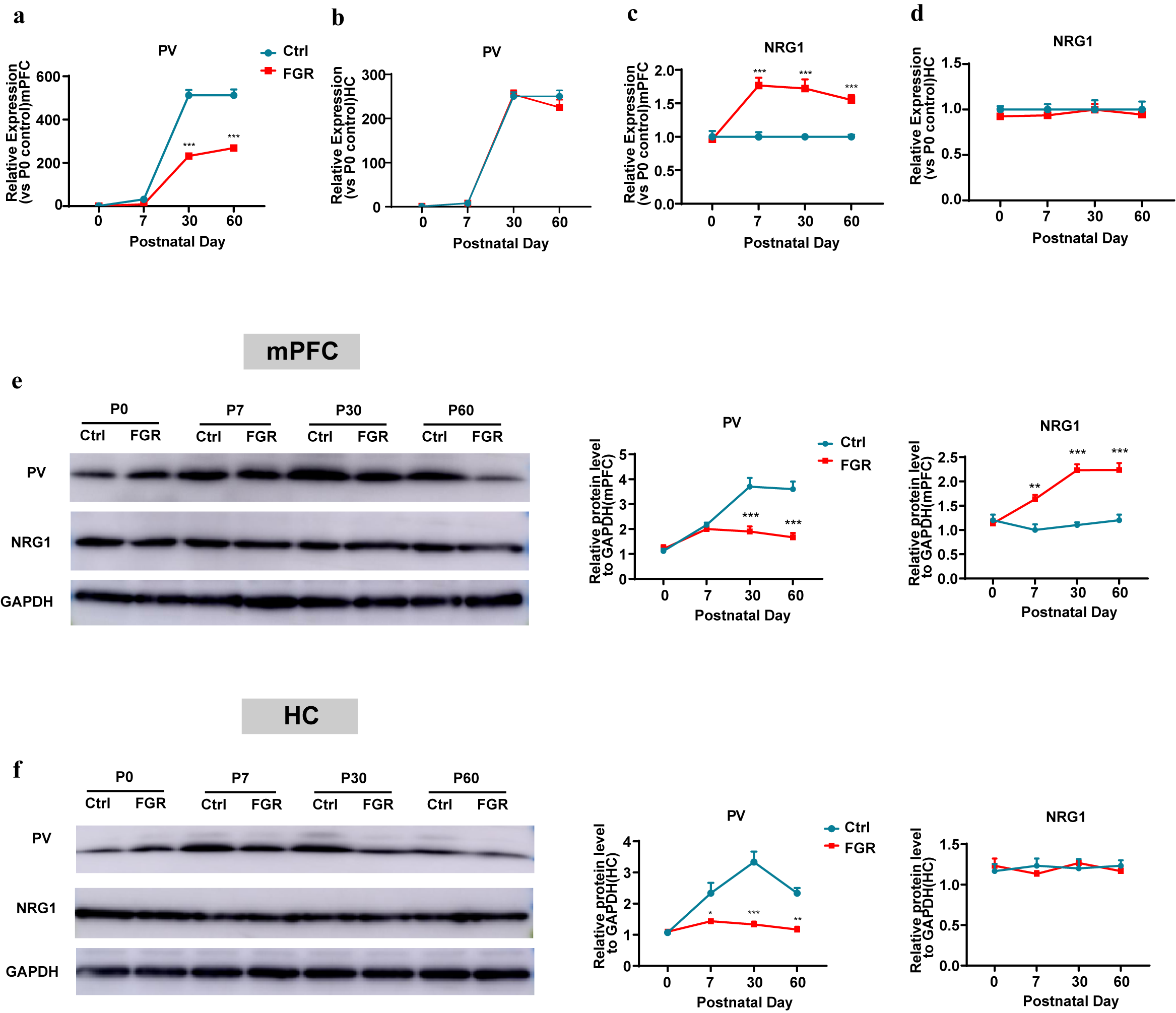


**Supplementary Figure 2 The postnatal dynamic mRNA and protein expression of PV and NRG1 during P0-P60.** (**a-d**) QPCR analysis of the mRNA level of PV and NRG1 in mPFC and hippocampus during P0-60. n = 6 in each group. (**a**) The mRNA level of PV in mPFC. One-way ANOVA, P30: Ctrl vs FGR, *P* < 0.001. P60: Ctrl vs FGR, *P* < 0.001. (**b**) The mRNA level of PV in hippocampus. (**c**) The mRNA level of NRG1 in mPFC. One-way ANOVA, P7: Ctrl vs FGR, *P* < 0.001. P30: Ctrl vs FGR, *P* < 0.001. P60: Ctrl vs FGR, *P* < 0.001. (**d**) The mRNA level of NRG1 in hippocampus. (**e,f**) Western blot analysis of the protein level of PV and NRG1 in mPFC and hippocampus during P0-60. n = 3 in each group. (**e**) The protein level of PV and NRG1 in mPFC. One-way ANOVA, P30-PV: Ctrl vs FGR, *P* < 0.001. P60-PV: Ctrl vs FGR, *P* < 0.001. P7-NRG1: Ctrl vs FGR, *P* = 0.003. P30-NRG1: Ctrl vs FGR, *P* < 0.001. P60-NRG1: Ctrl vs FGR, *P* < 0.001. (**f**) The protein level of PV and NRG1 in hippocampus. One-way ANOVA, P7-PV: Ctrl vs FGR, *P* = 0.02. P30-PV: Ctrl vs FGR, *P* < 0.001. P60-PV: Ctrl vs FGR, *P* < 0.001. The results are expressed as the mean ± SD.; **P* < 0.05, ***P* < 0.01 and ****P* < 0.001.
