# Supplementary material for: NRG1 knockdown rescues PV interneuron GABAergic maturation deficits and schizophrenia behaviors in fetal growth restriction mice
Source: Cell Death Discov. 2022 Dec 2;8:476. doi: 10.1038/s41420-022-01271-3 (PMC9718849; doi:10.1038/s41420-022-01271-3)
Supplement: Supplementary file 2 — Full and uncropped western blots [file 41420_2022_1271_MOESM2_ESM.docx]

**Supplementary Materials**

**Original western blots**

**NRG1 knockdown rescues PV interneuron GABAergic maturation deficits and schizophrenia behaviors in fetal growth restriction mice**

Jianfeng Dong^1^, Wen Chen^1^, Nana Liu^1^, Shujuan Chang^1^, Wei Zhu^1^, Jiuhong Kang^1*^

**Supplementary Data 1**


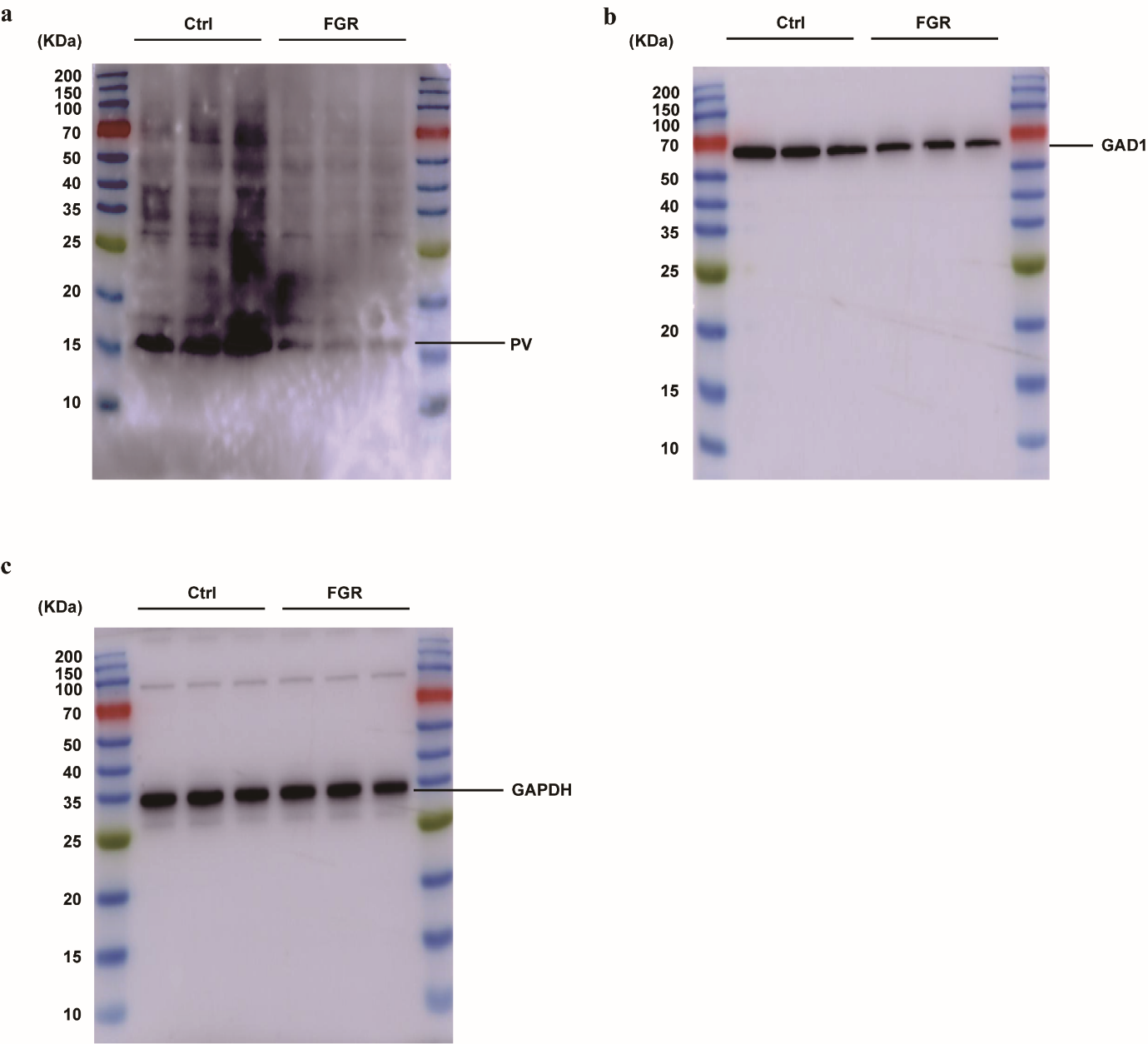


**Whole membrane image** **1**. **a,b,c** PV, GAD1 and GAPDH protein level of mPFC region, n = 3 in each group.

**Supplementary Data 2**


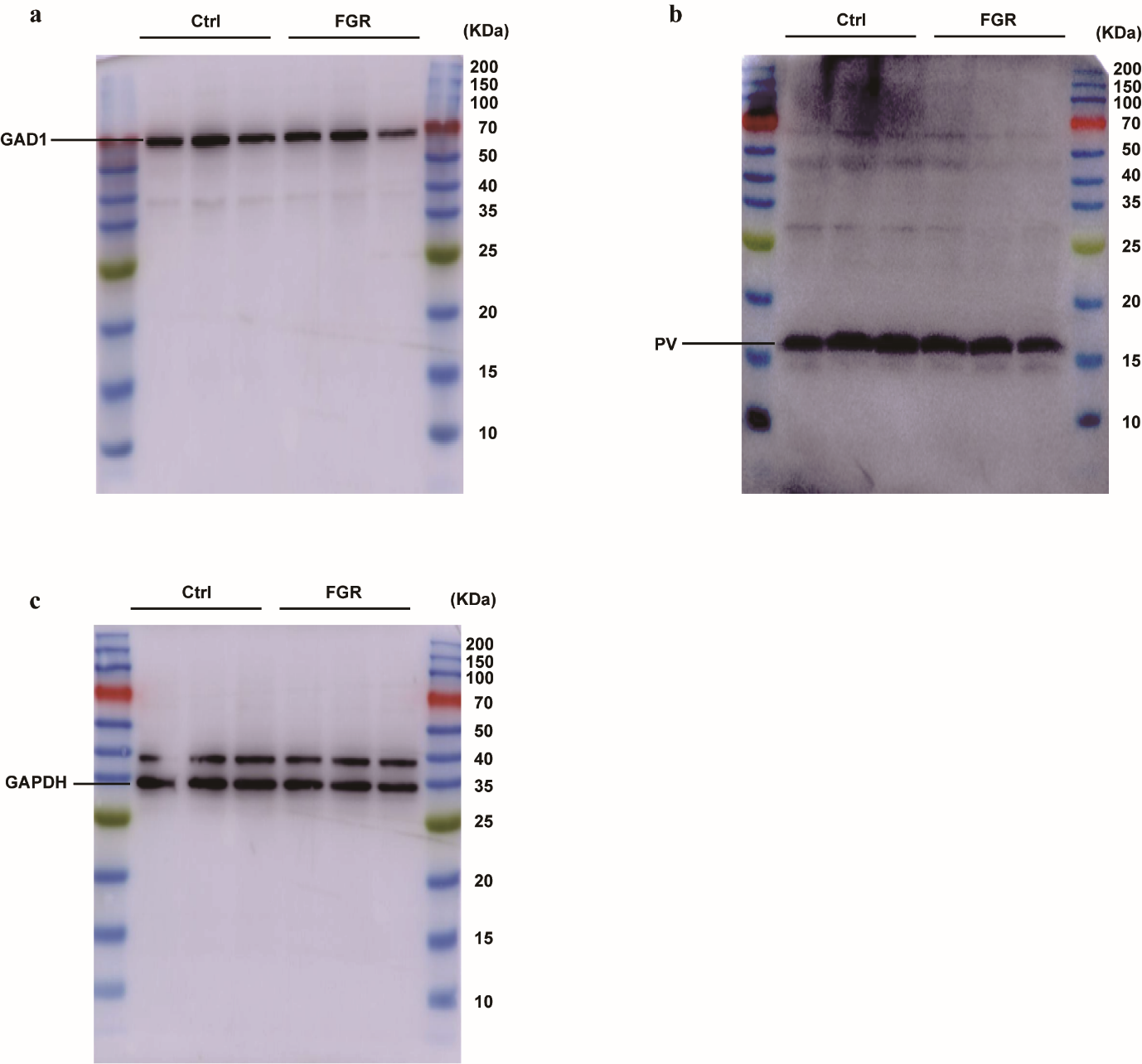


**Whole membrane image** **2**. **a,b,c** PV, GAD1 and GAPDH protein level of hippocampus region, n = 3 in each group.

**Supplementary Data 3**

**
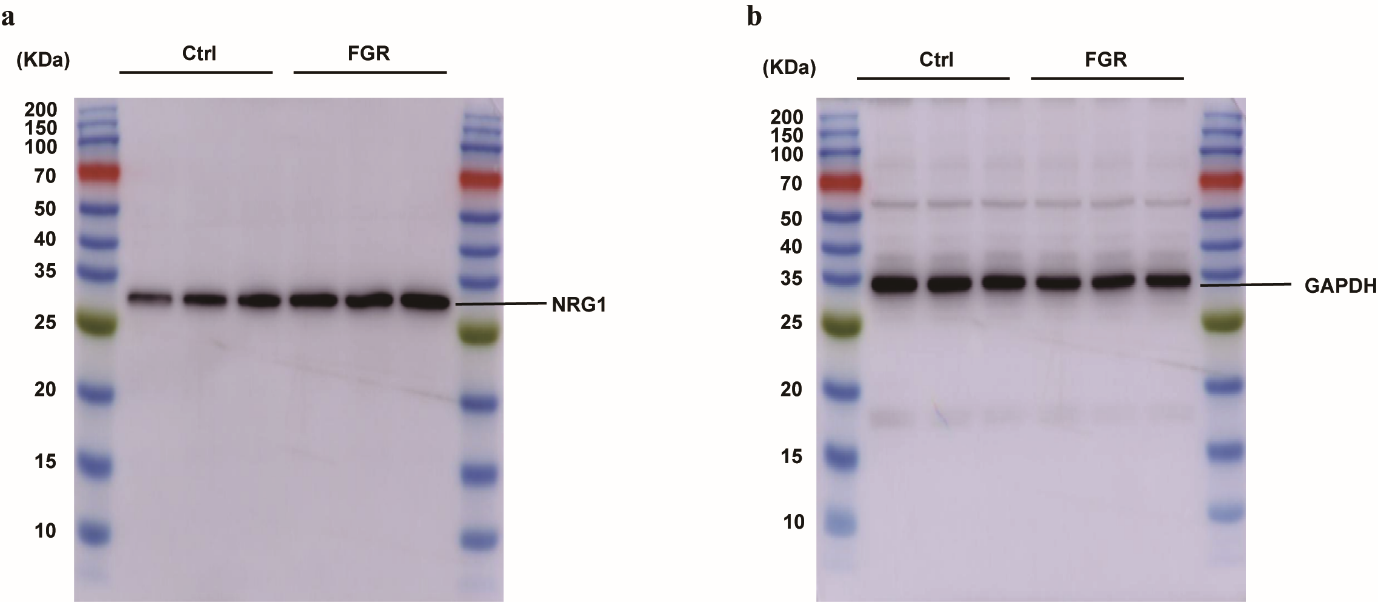
**

**Whole membrane image** **3**. **a,b** NRG1 and GAPDH protein level of mPFC region, n = 3 in each group.

**Supplementary Data 4**


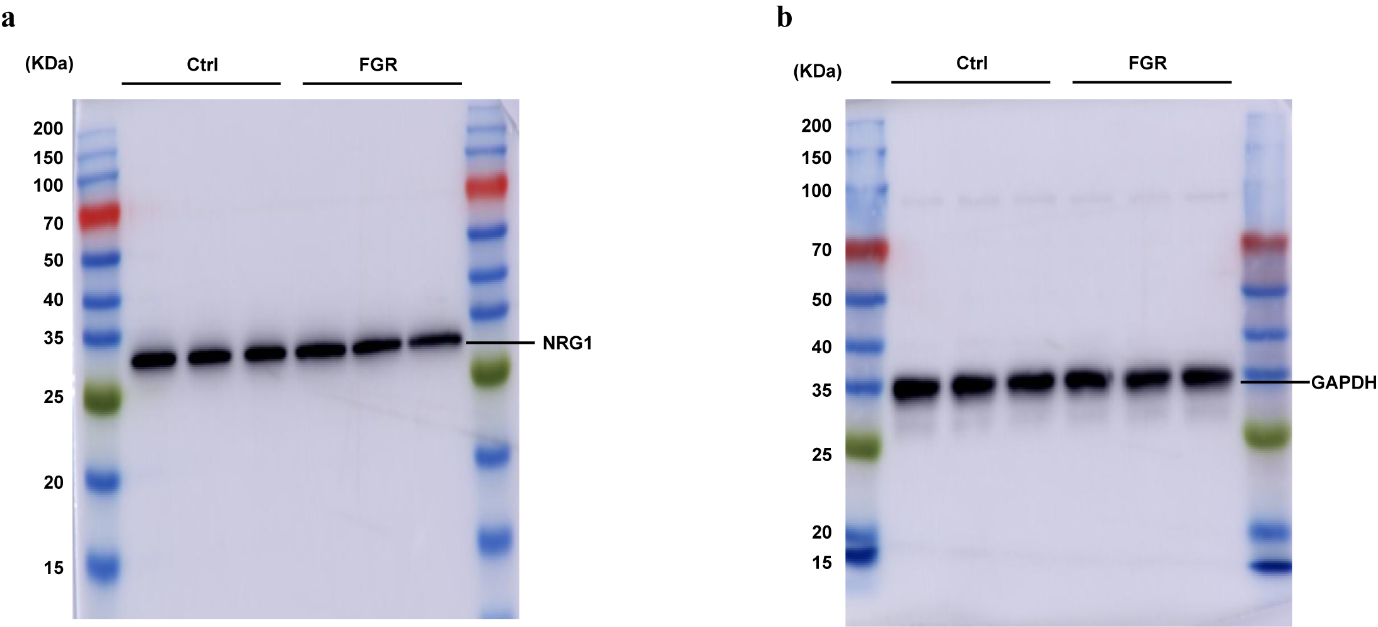


**Whole membrane image** **4**. **a,b** NRG1 and GAPDH protein level of hippocampus region, n = 3 in each group.

**Supplementary Data 5**


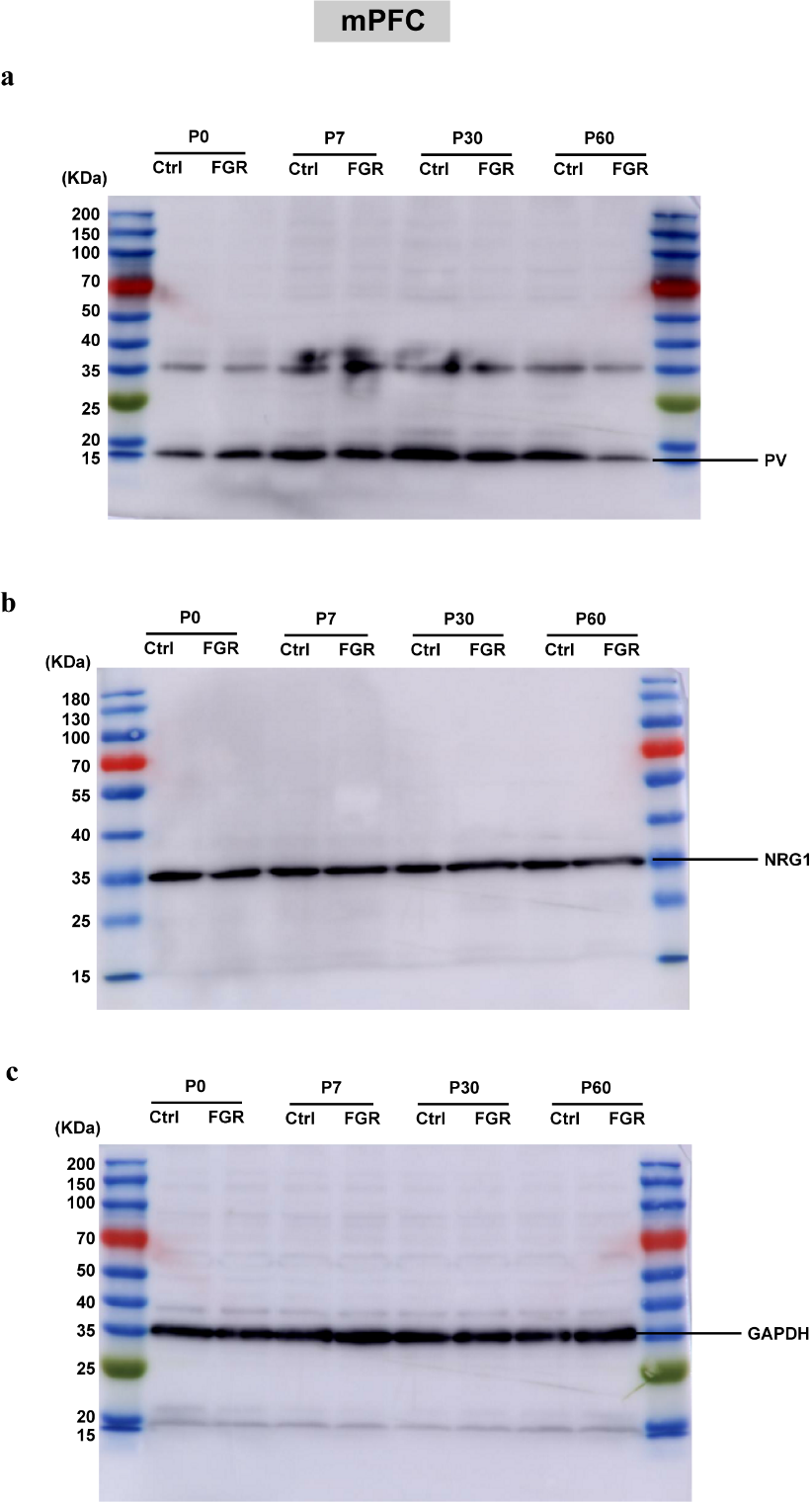


**Whole membrane image** **5**. **a-c** PV, NRG1 and GAPDH protein level of Ctrl and FGR mice mPFC , n = 3 in each group.

**Supplementary Data 6**


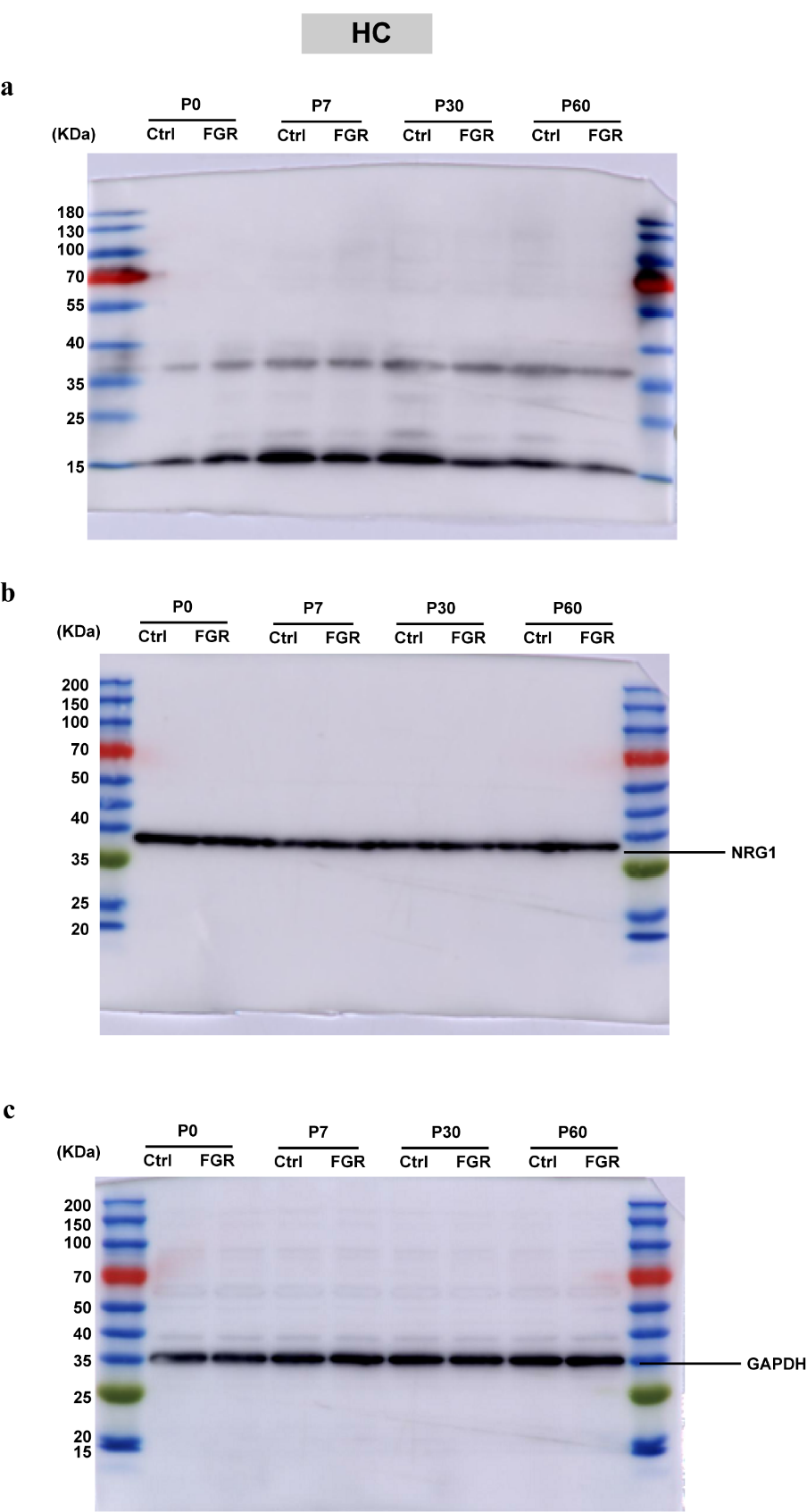


**Whole membrane image** **6**. **a-c** PV, NRG1 and GAPDH protein level of Ctrl and FGR mice hippocampus, n = 3 in each group.

**Supplementary Data 7**

**
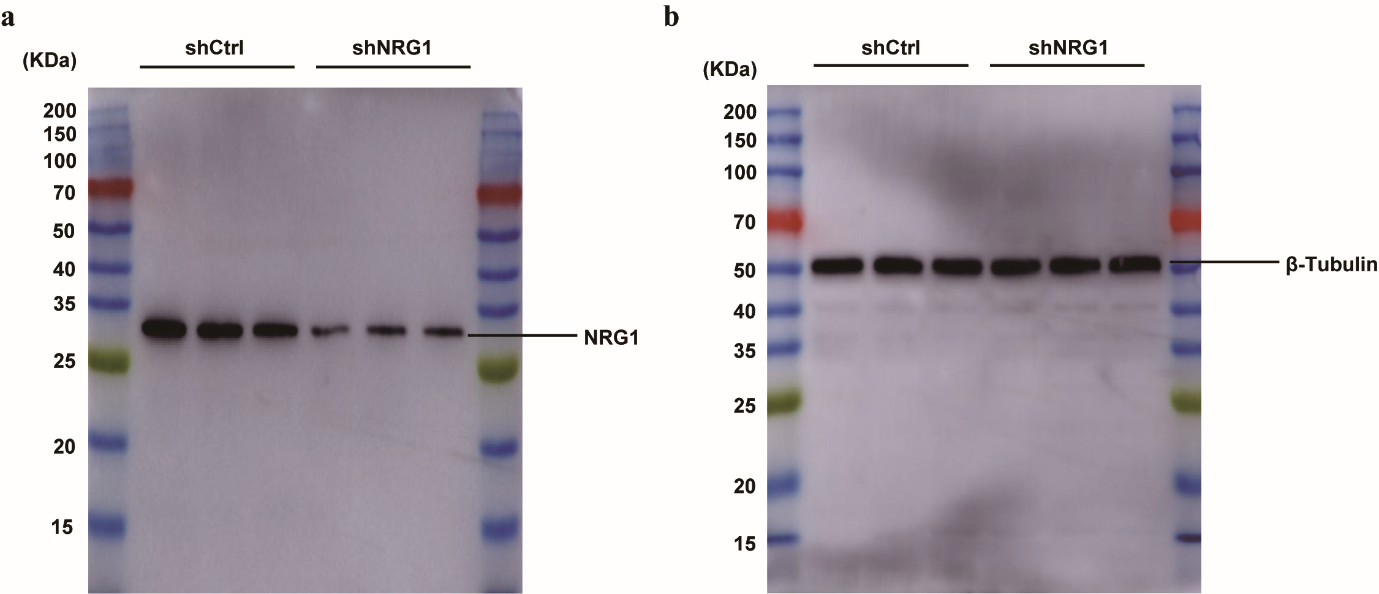
**

**Whole membrane image** **7**. **a,b** NRG1 and β-Tubulin protein level of shCtrl and shNRG1 groups, n = 3 in each group.

**Supplementary Data 8**


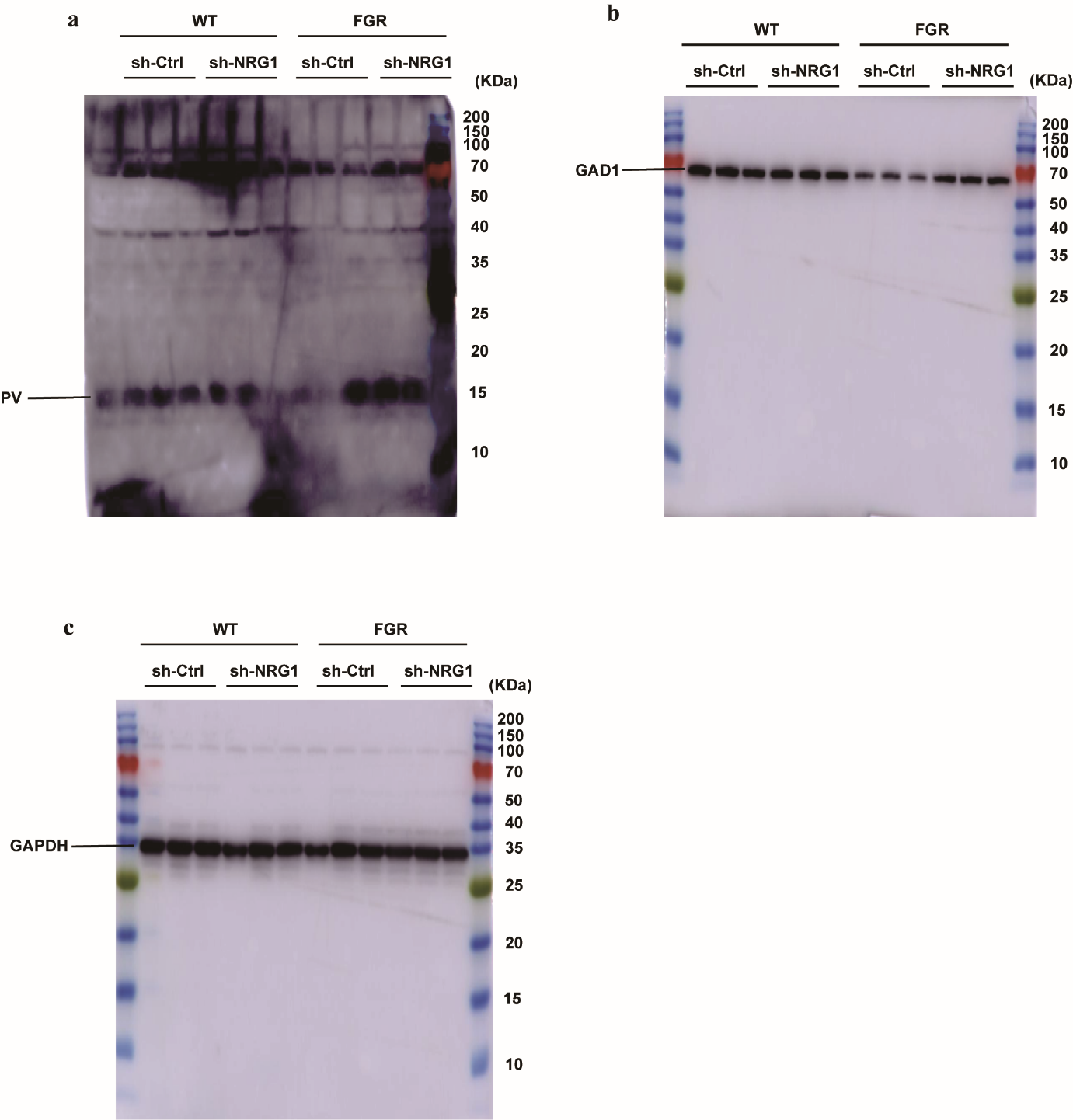


**Whole membrane image** **8**. **a,b,c** PV, GAD1 and GAPDH protein level of WT-shCtrl, WT- shNRG1, FGR-shCtrl and FGR-shNRG1 mice, n = 3 in each group.
